# The Toll-Like Receptor 2 Ligand Pam2CSK4 Activates Platelet Nuclear Factor-κB and Bruton’s Tyrosine Kinase Signaling to Promote Platelet-Endothelial Cell Interactions

**DOI:** 10.3389/fimmu.2021.729951

**Published:** 2021-08-30

**Authors:** Iván Parra-Izquierdo, Hari Hara Sudhan Lakshmanan, Alexander R. Melrose, Jiaqing Pang, Tony J. Zheng, Kelley R. Jordan, Stéphanie E. Reitsma, Owen J. T. McCarty, Joseph E. Aslan

**Affiliations:** ^1^Knight Cardiovascular Institute and Division of Cardiology, School of Medicine, Oregon Health & Science University, Portland, OR, United States; ^2^Department of Biomedical Engineering, School of Medicine, Oregon Health & Science University, Portland, OR, United States; ^3^Division of Hematology and Medical Oncology, School of Medicine, Oregon Health & Science University, Portland, OR, United States; ^4^Department of Chemical Physiology and Biochemistry, School of Medicine, Oregon Health & Science University, Portland, OR, United States

**Keywords:** platelets, Pam2CSK4, TLR2, adhesion, BTK

## Abstract

Circulating platelets establish a variety of immunological programs and orchestrate inflammatory responses at the endothelium. Platelets express the innate immunity family of Toll-like receptors (TLRs). While TLR2/TLR1 ligands are known to activate platelets, the effects of TLR2/TLR6 ligands on platelet function remain unclear. Here, we aim to determine whether the TLR2/TLR6 agonists Pam2CSK4 and FSL-1 activate human platelets. In addition, human umbilical vein endothelial cells (HUVECs) and platelets were co-cultured to analyze the role of platelet TLR2/TLR6 on inflammation and adhesion to endothelial cells. Pam2CSK4, but not FSL-1, induced platelet granule secretion and integrin α_IIb_β_3_ activation in a concentration-dependent manner. Moreover, Pam2CSK4 promoted platelet aggregation and increased platelet adhesion to collagen-coated surfaces. Mechanistic studies with blocking antibodies and pharmacologic inhibitors demonstrated that the TLR2/Nuclear factor-κB axis, Bruton’s-tyrosine kinase, and a secondary ADP feedback loop are involved in Pam2CSK4-induced platelet functional responses. Interestingly, Pam2CSK4 showed cooperation with immunoreceptor tyrosine-based activation motif (ITAM)-mediated signaling to enhance platelet activation. Finally, the presence of platelets increased inflammatory responses in HUVECs treated with Pam2CSK4, and platelets challenged with Pam2CSK4 showed increased adhesion to HUVECs under static and physiologically relevant flow conditions. Herein, we define a functional role for platelet TLR2-mediated signaling, which may represent a druggable target to dampen excessive platelet activation in thrombo-inflammatory diseases.

## Introduction

Toll-like receptors (TLRs) belong to the pattern recognition receptors (PRRs) innate immunity family and play a central role in the initiation of the immune response and host defense (1). PRRs are expressed in most cellular types and sense pathogen-associated molecular patterns (PAMPs) and damage-associated molecular patterns (DAMPs), which are released upon tissue injury in a process known as sterile inflammation (2, 3). PAMPs and DAMPs are enriched in inflammatory *milieus* and may promote dysregulated TLRs responses, which has been suggested to contribute to the pathogenesis of atherosclerosis, acute coronary syndromes, stroke, viral myocarditis, sepsis, ischemia/reperfusion injury, and heart failure (4–7). Classical TLR ligands include bacterial lipopolysaccharide (LPS) for TLR4 (8), as well as bacterial wall acylated lipopeptides binding to TLR2/TLR1 and TLR2/TLR6 dimers (9, 10). TLR2 and TLR4 signaling *via* myeloid-differentiation factor 88 (MyD88) bifurcates into two main pathways, namely the nuclear factor (NF)-κB family and the mitogen-activated protein kinases (MAPKs) cascade, which subsequently initiates an inflammatory response by activating target genes related to cytokine expression, cell survival, and proliferation (1, 2, 11–13).

In addition to their roles as primary cellular effectors of hemostasis, platelets also regulate inflammation and immune responses (14, 15). Platelets secrete chemokines and cytokines with immunomodulatory functions, as well as antimicrobial peptides that regulate bacterial growth (16, 17). Platelets also express functional immune receptors, including all TLRs at the transcript and protein levels, and therefore are able to initiate a rapid immune response in the context of infection or sterile inflammation (18–20). However, dysregulated platelet immune function may also promote excessive inflammation and coagulation in a pathologic process recently termed “immunothrombosis” or “thromboinflammation”, which is characteristic of infective diseases such as sepsis and coronavirus disease-19 (21–26). In sepsis, platelet consumption and activation play a deleterious role, yet whether platelets are directly activated by bacterial motifs in infection remains largely unexplored (21, 27). Dysregulated platelet activation has also been related to the development of cardiovascular disease (28–31). Excessive platelet activation and platelet interactions with leukocytes and endothelial cells (ECs) drive inflammation and the development of vascular disorders, including atherosclerosis (32–35). For example, platelets can bind to and interact with ECs in a manner which contributes to exacerbated endothelial dysfunction in cerebrovascular inflammation (36).

Specific signaling pathways activated by TLRs and inflammatory ligands in platelets may provide a means of targeting thrombosis in inflammatory conditions without affecting hemostasis (19), yet the physiological and functional roles of TLRs in platelets remain unclear. The canonical TLR4 ligand LPS has been shown to promote and potentiate platelet activation *in vitro* (37, 38); while other studies have reported a decrease or absence in platelet activation in response to LPS (39). *In vivo* studies demonstrated that activation of platelet TLR4 promotes neutrophil extracellular trap formation in patients with sepsis (40). In addition to TLR4, platelets also express TLR2 and its coreceptors TLR1 and TLR6 (20). The triacylated lipopeptide and TLR2/TLR1 agonist, Pam3CSK4, induced platelet activation *via* phosphoinositide-3 kinase (PI3K) pathways in both *in vitro* and *in vivo* models (41–43), while another study reported roles for NF-κB in Pam3CSK4-induced platelet activation (44). Little has been reported regarding the effects of agonists activating the platelet TLR2/TLR6 complex. An elegant study by Biswas and colleagues demonstrated a contribution for TLR2/TLR6 on platelet activation in hyperlipidemia by synergizing with CD36 (31). It is noteworthy that Blair *et al*. reported inconsistent platelet aggregation in response to Pam2CSK4 (41). These results are in contrast with other studies showing that the TLR2/TLR6 activators macrophage-activating lipopeptide 2 (MALP-2) and fibroblast-stimulating ligand 1 (FSL-1) failed to promote platelet activation in purified systems (45–47).

Here, we define how platelets respond to the TLR2/TLR6 activators Pam2CSK4 and FSL-1 *in vitro* and *ex vivo*. We found that Pam2CSK4, but not FSL-1, potently induced a set of platelet responses *via* TLR2/NF-κB and Bruton’s-tyrosine kinase (BTK). In addition, we found that activation of platelet TLR2 increased inflammatory responses of HUVECs and promoted adhesion of platelets to HUVECs under static and flow conditions.

## Materials and Methods

### Reagents

Soluble collagen was from Corning (Corning, NY, USA). Prostaglandin I_2_ (PGI_2_) was from Cayman Chemical (Ann Arbor, MI, USA). Crosslinked collagen-related peptide (CRP-XL) was from R. Farndale (CambCol Laboratories, Cambridge University, UK). CHRONO-LUME^®^ detection reagent was from Chrono-Log Corporation (Havertown, PA, USA; #395). Pam2CSK4 and Pam3CSK4 were from Invivogen (#tlrl-pm2s-1 and #tlrl-pms). Tumor necrosis factor-α (TNF-α, #210-TA-020) was from R&D Systems. FSL-1 was from Tocris (#6011). Anti-TLR2 (#maba2-htlr2), anti-TLR6 (magb-htlr6) and control IgG (mabg1-ctrlm) and IgA (maba2-ctrl) antibodies were from Invivogen. All other reagents were from Sigma-Aldrich or previously named sources (48).

#### Antisera

Flow cytometry antibodies CD62P-APC and PAC1-FITC were from BioLegend (San Diego, CA, USA) and Becton Dickinson (Franklin Lakes, NJ, USA) respectively. TLT1 was from R&D Systems (#FAB2394G). Akt S_473_ (9271), NF-κB p65 S_536_ (3033), BTK Y_551_ (18805), DAPP1 Y_139_ (13703), PLCγ2 Y_1217_ (3871), Syk Y_525_ (2711), phosphorylated Akt substrate (9614), phosphorylated MAPK substrate (2325), and phosphorylated PKC substrate (6967) antibodies were purchased from Cell Signaling Technology (Danvers, MA, USA). 4G10 was from Sigma Millipore (#05-321). Tubulin (#T6199) antibody was from Sigma-Aldrich. Anti CD41-FITC antibody was from BD Biosciences (#555466).

#### Inhibitors

C29 (#HY-100461) was from MedchemExpress. BAY11-7082 (#tlrl-b82) was from Invivogen. Acalabrutinib and ibrutinib were from Selleck chem (#S8116 and #S2680 respectively).

### Platelet Isolation

Human venous blood was drawn from healthy adult male and female volunteers by venipuncture into 3.8% sodium citrate (1:10, v/v) following an Institutional Review Board protocol approved by Oregon Health & Science University, as previously described (49). Blood was centrifuged for 20 min at 200×*g* at room temperature (rt) and the platelet-rich plasma (PRP) was collected. PRP was centrifuged at 1000×*g* for 10 min in the presence of the platelet inhibitor prostaglandin (PG)I_2_ at 0.1 µg/ml. The platelet pellet was resuspended in HEPES/Tyrode buffer (HT; 129 mM NaCl, 0.34 mM Na_2_HPO_4_, 2.9 mM KCl, 12 mM NaHCO_3_, 20 mM HEPES, 5 mM glucose, 1 mM MgCl_2_; pH 7.3) containing 10% acid citrate dextrose buffer. Finally, platelets were centrifuged in the presence of PGI_2_ at 1000×g for 10 min, resuspended in HT, and diluted in HT at the indicated concentrations for each assay.

### Static Adhesion Assay

Glass coverslips (12 mm round, #1.5 thickness, 12-545-102) were coated with soluble collagen at 50 µg/ml for 1 h at rt, followed by surface blocking with denatured fatty-acid free bovine serum albumin (BSA) at 5 mg/ml (1h at rt). 10 µg/ml of Pam2CSK4 was added to platelets and cells were incubated for 45 min at 37°C onto the collagen coverslips. Platelets were then washed 3 times with phosphate buffered saline (PBS) and fixed with 4% paraformaldehyde (PFA) for 15 min at rt. After 3 washes with PBS, coverslips were mounted and imaged using Kohler-illuminated Nomarski differential interference contrast (DIC) optics with a Zeiss 63× oil immersion lens (28). Platelets were imaged using Slidebook 5.0 software (Intelligent Imaging Innovations). Data are expressed as the number of platelets per imaged field (14,587 μm^2^), which was manually calculated using ImageJ.

### Platelet Aggregation

300 μl of platelets at 3×10^8^/ml were stimulated with the indicated ligands and monitored under continuous stirring at 1200 rpm at 37°C by measuring changes in light transmission in a 490-4+4 aggregometer (Chrono Log Corporation). Data are presented as the average of total percentage of aggregation for 4 different experiments.

### Platelet Flow Cytometry

Washed platelets (5×10^7^/ml) were stimulated with CRP-XL, or the TLR2/TLR6 ligands FSL-1 and Pam2CSK4 for 25 min in the presence of CD62P-APC and PAC1-FITC antibodies (1:25). For mechanistic studies, platelets were pre-incubated with the indicated inhibitors or vehicle (equivalent dose of DMSO) for 10 min at 37°C before activation. On another subset of experiments, TLT1-FITC at 1:25 was used. For some experiments, citrated whole blood was diluted 1:5 in HT and platelets were gated based on SSC and FSC characteristics. PAC1 and CD62P staining in the platelet gate was measured as mentioned above. Washed platelet and whole blood samples were fixed with 2% PFA, diluted in PBS and analyzed with a BD FACSCanto II flow cytometer. A total of 20000 events were recorded at low acquisition speed. Data were analyzed using FlowJo software and presented as mean fluorescence intensity (MFI).

### Dense Granule Secretion Assay

ATP release was used to measure dense granule secretion followed by an ATP-luciferin-luciferase luminescence reaction. Platelets (2×10^8^/ml; 80 µl) were stimulated with the indicated agonists in a Corning Costar flat bottom 96-well plate and the CHRONO-LUME reagent (10 μl) was immediately added and luminescence measured using an Infinite M200 spectrophotometer (Tecan). For mechanistic studies, platelets were pre-incubated with the indicated inhibitors or vehicle for 10 min at 37°C before activation with Pam2CSK4. Data are expressed as total luminescence arbitrary units (a.u.).

### HUVEC Culture and HUVEC-Platelet Interactions

HUVECs (ATCC, Manassas, VA, USA) were grown to confluence on 0.1% gelatin-coated T75 flasks with Vasculife^®^ medium kit (#LL-0003; Lifeline Cell Technology). For experiments, HUVECs were cultured onto 12-well plates until reaching confluence and stimulated with Pam2CSK4 (10 µg/ml), Pam3CSK4 (10 µg/ml) or TNF-α (1 ng/ml) as indicated. Before stimulation, cells were serum-starved for 2 h in serum-free medium (SFM). Cells between passages 4 and 8 were used for all the experiments. For HUVECs-platelet co-culture, 250 µl of platelets (2×10^8^/ml) were mixed with 250 µl of SFM and co-cultured with HUVECs for 4 hours. Supernatants were collected and assayed by ELISA. Adhered platelets were thoroughly washed with SFM and HUVECs lysates prepared as indicated below.

### TLR2 Detection by Flow Cytometry

HUVECs were cultured onto 6-well plates until reaching confluence and stimulated with Pam2CSK4 (10 µg/ml) or TNF-α (1 ng/ml) in the presence or absence of platelets (2x10^8^/ml) for 4 h at 37°C. Then, cells were washed thoroughly with PBS and HUVECs harvested using Accutase™ (ThermoFisher Scientific; #A1110501). Cells were centrifuged, resuspended and stained with a TLR2-FITC antibody (ThermoFisher Scientific, #11-9922-42; 1:20) or the corresponding IgG-FITC isotype control (ThermoFisher Scientific, #11-4724-81; 1:20) for 1 h at 4°C. Samples were washed, fixed with 2% PFA, diluted in PBS and analyzed with a BD FACSCanto II flow cytometer. Data were analyzed using FlowJo software and presented as mean fluorescence intensity (MFI).

### Western Blot

Platelets (1×10^9^/ml) were stimulated with the indicated ligands at 37°C. Platelet lysates were prepared in Laemmli sample buffer supplemented with 200 mM DTT. Platelet lysates were separated by SDS-PAGE and transferred to nitrocellulose membranes. For HUVECs experiments, cells were seeded in 12-well platelets overnight, co-cultured with platelets and lysed with m-Per mammalian protein extraction reagent (ThermoFisher Scientific, #78501). The bicinchoninic acid assay (BCA; ThermoFisherScientific; #23225) was performed to achieve equal loading within the samples. Primary antibodies were incubated overnight at 4°C and secondary antibodies for 1 h at rt. Films were scanned and blots representative of 3-4 independent experiments are shown.

### Cytokine Secretion by ELISA

Supernatants from the co-culture experiments were initially centrifuged at 1000×g for 5 min at rt to remove platelets. The supernatant was centrifuged again at 12000×g for 10 min to remove debris, diluted and assayed for IL secretion. For platelet secretome studies, platelets were stimulated with the indicated ligands for 45 min at 37°C and then pelleted by centrifugation at 1000×g for 5 min. The supernatant was collected, centrifuged at 13000×g for 5 min to remove debris and assayed for IL secretion without dilution. Secretion of interleukin (IL)-8 (R&D systems; #D800C) and IL-6 (R&D systems; #D6050) was assessed following manufacturer’s protocol. Technical duplicates were performed and data expressed as pg/ml of total protein.

### Permeability in Transwell Assay

HUVECs were seeded to confluence in gelatin-coated upper membranes of Transwell devices (0.4 µm, Corning, #3450). Cells were then stimulated in complete media for 4 h at 37°C with Pam2CSK4 (10 µg/ml) or TNF-α (1 ng/ml) in the presence or absence of platelets (final concentration of 1x10^8^/ml). Cells were then washed 3 times with media to remove platelets. To analyze HUVECs permeability to BSA, media containing Evans Blue dye (0.67 mg/ml) and 4% BSA was added to the upper chamber for 30 min at 37°C. Samples from the lower chamber were collected and absorbance at 650 nm was measured using a spectrophotometer (Tecan) as previously described (50).

### Platelet-HUVEC Adhesion Experiments

HUVECs were seeded to confluence into Ibidi µ-Slide VI 0.1 chambers (Ibidi, #80666), serum starved for 2 h and stimulated with TNF-α at 1 ng/ml or Pam2CSK4 (10 µg/ml) for 4 h when indicated. For static and flow adhesion assays, platelets (2×10^8^/ml) were stimulated with Pam2CSK4 (10 µg/ml) and stained with a α-CD41-FITC antibody (1:10) for 10 min at 37°C prior to co-culture with HUVECs. For static assays, platelets (50 µl) were diluted 1:1 in SFM and incubated with HUVECs for 10 min at 37°C. For flow adhesion experiments, the Ibidi chamber was connected to a syringe-coupled pump system. Platelets were flowed for 5 min at a flow rate of 18.69 µl/min (200 s^-1^ shear rate). After the adhesion experiments, non-adhered cells were removed by washing 3 times with PBS and cells were fixed for 15 min with 4% PFA. After washing 3 times with PBS, HUVECs were permeabilized and stained for nuclei with PBS containing 0.1% SDS and 1:1000 of Hoesch dye (Invitrogen, #3570). Cells were imaged with a 20X objective on an inverted microscope (Zeiss Axiovert 200M, Carl Zeiss). 5 random images were taken per field and the percentage of area covered by platelet CD41 staining was manually calculated using ImageJ.

### Statistical Analysis

Two group data presented in the study followed normal distribution and were analyzed performing a two-tailed Student’s t-test. For three or more groups, data were analyzed by one-way ANOVA with a Tukey post-hoc test. Statistical significance was considered for p<0.05. Statistic calculations were performed using GraphPad PRISM 9 (San Diego, CA, USA). For all the experiments, n indicates the number of independent experiments performed with cells isolated from different donors.

## Results

### Pam2CSK4, but Not FSL-1, Activates Platelets *In Vitro* and *Ex Vivo*

**Figure 1:**
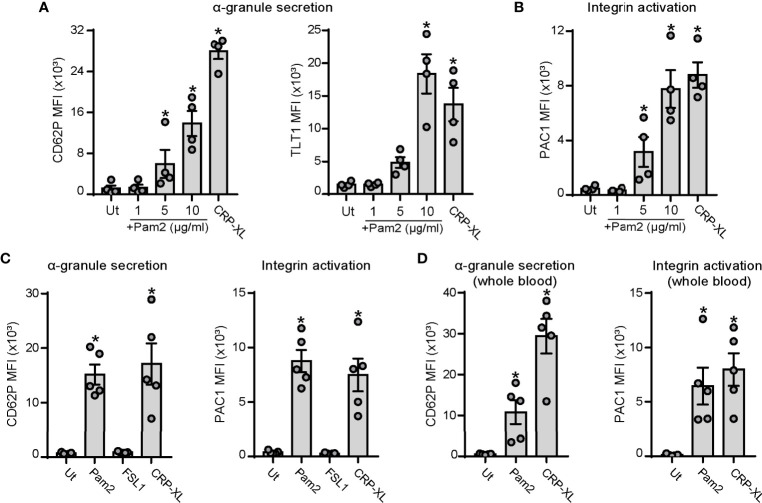
Pam2CSK4 induces platelet activation in purified cells and in citrated whole blood. **(A-C)** Washed platelets (5×10^7^/ml) were activated with the indicated ligands in the presence of fluorophore-conjugated antibodies against P-selectin (CD62P-APC), TLT1 (TLT1-FITC) or the activated conformation of human integrin α_IIb_β_3_ (PAC1-FITC) at 1:25 and subsequently analyzed by flow cytometry. CRP-XL was used as positive control (5 µg/ml). **(A, B)** Dose response for Pam2CSK4-mediated activation of human washed platelets; n=4 independent experiments. **(C)** Comparison between Pam2CSK4 (10 µg/ml) and another TLR2/TLR6 ligand, FSL-1 (10 µg/ml); n=5 independent experiments. **(D)** Citrated whole blood was diluted 1:5 in HT and activated with Pam2CSK4 at 10 µg/ml or CRP-XL at 2 µg/ml. CD62P-APC and PAC1-FITC staining was measured in the platelet gate; n=5 independent experiments. Data are presented as total mean fluorescence intensity (MFI). Pam2 indicates Pam2CSK4. * indicates statistical significance (p<0.05) compared to untreated (Ut). Error bars indicate standard error of the mean (SEM).

Platelet activation and interactions with ECs is a common hallmark of infective diseases such as sepsis (21, 51) and viral infections (52). We first sought to determine whether the bacterial-like motif and TLR2/TLR6 ligand, Pam2CSK4, activates platelets. We measured platelet α-granule secretion and integrin activation by staining for P-selectin (CD62P), Trem-like transcript 1 (TLT1) (53) and PAC1 and analysis by flow cytometry. Crosslinked collagen-related peptide (CRP-XL), which specifically engages the glycoprotein VI (GPVI) and Fc receptor-γ chain complex, was used as positive control. Incubation of washed human platelets with Pam2CSK4 promoted P-selectin and TLT1 exposure in a concentration-dependent manner, reaching highest levels at a concentration of 10 µg/ml (**Figure 1A**). Platelet activation was also assessed by PAC1 staining, which binds specifically to the activated form of the integrin α_IIb_β_3_. Consistent with α-granule secretion data, Pam2CSK4 induced integrin α_IIb_β_3_ activation in a concentration-dependent manner (**Figure 1B**). Next, we analyzed whether FSL-1, another diacylated lipopeptide and TLR2/TLR6 ligand, promoted platelet activation in a similar manner than Pam2CSK4. However, FSL-1 failed to promote either α-granule secretion and integrin activation at doses ranging from 1 to 20 µg/ml, as assessed by flow cytometry (**Figure 1C** and **Supplementary Figures 1A, B**). Finally, we examined whether Pam2CSK4 promoted platelet activation under physiologically-relevant conditions in whole blood. PAC1 and P-selectin staining demonstrated to Pam2CSK4 activates platelets also in whole blood (**Figure 1D**), whereas FSL-1 did not promote platelet activation under these conditions (**Supplementary Figures 1C, D**). Altogether, these results demonstrate that Pam2CSK4 is a potent platelet activator, and that specific TLR2/TLR6 ligands (i.e. Pam2CSK4 *vs* FSL-1) may differentially affect platelet functional responses.

### Effects of Pam2CSK4 on Aggregation and Signaling in Platelets

**Figure 2:**
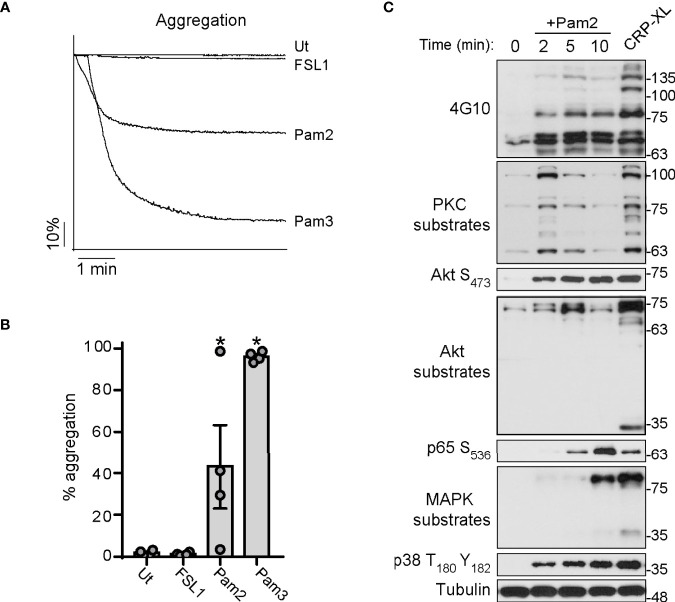
Pam2CSK4 is weak to induce aggregation but triggers the activation of several signaling pathways in platelets. **(A, B)** Washed platelets (3×10^8^/ml) were stimulated with the indicated ligands and monitored under continuous stirring at 1200 rpm at 37°C for 6 minutes in an aggregometer. All the ligands were used at 10 µg/ml. **(A)** Traces of a representative experiment are shown. **(B)** Data are presented as the average of total percentage of aggregation for n=4 different experiments. **(C)** Human washed platelets (1x10^9^/ml) were stimulated with Pam2CSK4 (10 µg/ml) for the indicated times or CRP-XL at 2 μg/ml for 5 min. Cell lysates were analyzed by Immunoblot for the indicated antibodies. Representative blots from n=4 independent experiments are shown. * indicates statistical significance (p<0.05) compared to Untreated (Ut). Error bars indicate standard error of the mean (SEM).

We next aimed to determine whether Pam2CSK4 and FSL-1 induce platelet aggregation, using Pam3CSK4 as positive control. As shown in **Figures 2A, B**, Pam3CSK4 promoted robust and maximal platelet aggregation in all experiments. In contrast, stimulation of platelets with Pam2CSK4 results in a heterogeneous, donor-dependent aggregation response ranging from almost-null to almost-full platelet aggregation (**Figures 2A, B**). In contrast, platelets did not aggregate in response to FSL-1 treatment (**Figures 2A, B**).

Then, we sought to analyze signaling events in response to Pam2CSK4 treatment. Washed platelets were stimulated with Pam2CSK4 over a time course prior to lysis and Western blot analysis for protein phosphorylation markers in platelet activation pathways, where CRP-XL served as a positive control. As seen in **Figure 2C**, Pam2CSK4 induced a robust increase in tyrosine phosphorylation events in platelets as demonstrated with a 4G10 antibody. Pam2CSK4 also activated two of the main signal transduction pathways in platelets, namely the protein kinase C (PKC) and the protein kinase B (Akt) pathways, which is demonstrated by the increase in phosphorylation at the Akt S_473_ site as well as of downstream substrates (**Figure 2C**). Consistent with canonical signal transduction downstream of TLR2 (54), Pam2CSK4 promoted the activation of p65-NF-κB S_536_ and the MAPKs complex, as shown by the increase of phosphorylation in MAPKs substrates and p38 T_180_Y_182_. These data demonstrate that Pam2CSK4 induces platelet aggregation and triggers signaling events consistent with the activation of TLRs pathways and key nodes on platelet function.

### The TLR2/TLR6/NF-kB Axis and an ADP Feedback Loop Mediate Pam2CSK4 Responses

**Figure 3:**
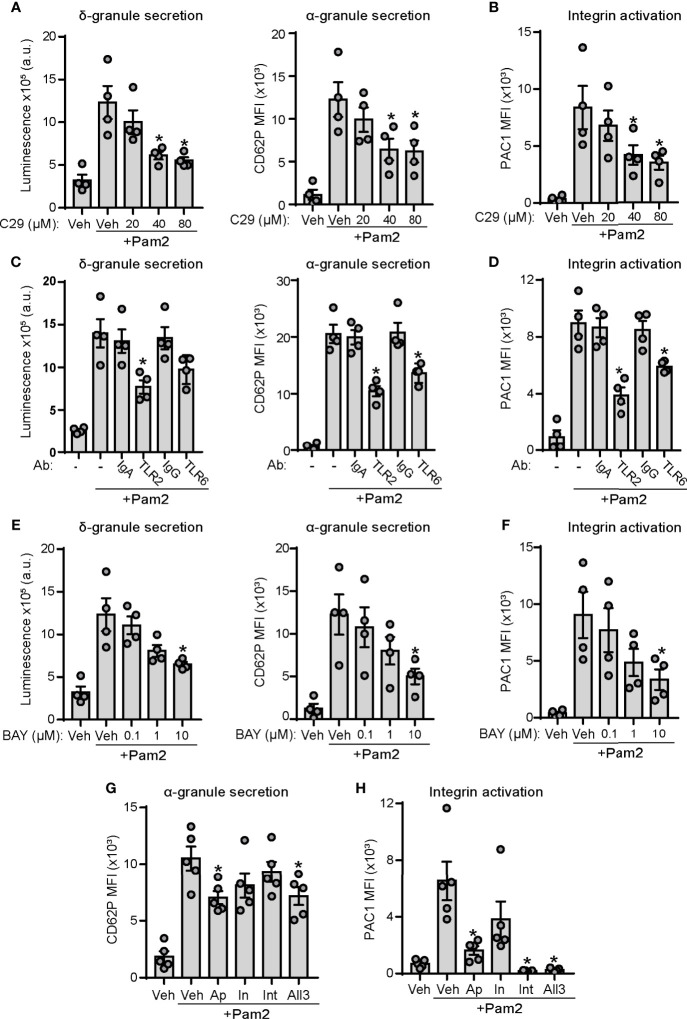
TLR2/TLR6/NF-κB axis and an ADP-positive feedback loop play a role in Pam2CSK4-induced platelet function. **(A–H)** Washed human platelets were preincubated with the indicated blocking antibodies (25 µg/ml; 30 min), inhibitors or vehicle (10 min) at 37°C before activation with Pam2CSK4 (10µg/ml) and analysis of platelet granule secretion and integrin activation. **(A, C, E)** Platelets (2×10^8^/ml) were incubated with a luciferin luciferase reagent to measure ATP release and dense granule secretion. Data are expressed as raw mean luminescence (a.u.); n=4 experiments. **(B, D, F–H)** Platelets (5×10^7^/ml) were pre-treated and activated as above in the presence of fluorophore-conjugated antibodies against P-selectin (CD62P-APC) or the activated conformation of human integrin α_IIb_β_3_ (PAC1-FITC) and subsequent analysis by flow cytometry; n=4-5 experiments. Data are presented as total mean fluorescence intensity (MFI). * indicates statistical significance (p<0.05) compared to Pam2CSK4 (- or Veh). BAY indicates BAY11-7082, a NF-κB inhibitor; Ap, 2 U/ml apyrase; In, 10 μM indomethacin; Int, 20 μg/ml integrilin. All 3 indicates combination of apyrase, indomethacin and integrilin at the doses previously indicated. Error bars indicate standard error of the mean (SEM).

To specify the contribution of TLR2 and TLR6 signaling in Pam2CSK4-mediated platelet activation, we performed pharmacological experiments with C29, a novel TLR2 inhibitor (55), as well as receptor blockade assays using blocking antibodies against TLR2 and TLR6. As shown in **Figure 3A**, preincubation of platelets with increasing doses of C29 partially inhibited dense granule secretion induced by Pam2CSK4. We found a similar effect of C29 on platelet α-granule secretion and integrin activation (**Figures 3A, B**). We next performed receptor blockade experiments. Control isotypes IgA and IgG did not affect the extent of platelet granule secretion or integrin activation induced by Pam2CSK4 (**Figures 3C, D**). In contrast, preincubation with a TLR2 blocking antibody significantly reduced platelet function in response to Pam2CSK4, whereas blocking TLR6 promoted milder but significant inhibition of platelet α-granule secretion and integrin activation (**Figures 3C, D**). Finally, to define the role of the NF-κB pathway in Pam2CSK4-mediated responses, we preincubated platelets with BAY11-7082, a p65 activation inhibitor previously characterized in platelets (56, 57), prior to stimulation with Pam2CSK4. Preincubation of platelets with BAY11-7082 reduced platelet responses to Pam2CSK4 in a concentration-dependent manner (**Figures 3E, F**), suggesting a role for NF-κB activation downstream of TLR2 in platelets.

Adenosine diphosphate (ADP) and thromboxane A2 (TxA2) positive feedback loops play a key role on platelet activation in response to hemostatic and inflammatory agonists (58), including Pam3CSK4 (47). In addition, integrin α_IIb_β_3_ outside-in signaling has been pointed out as a key mechanism for maintaining and amplifying platelet responses after binding of substrates to the activated form of the integrin (59). We next analyzed whether Pam2CSK4 responses are dependent on these secondary loops. Flow cytometry experiments demonstrated that addition of the ADP scavenger apyrase caused a mild but significant reduction in platelet α-granule secretion induced by Pam2CSK4 (**Figure 3G**). Moreover, integrin α_IIb_β_3_ activation was largely dependent on the ADP positive feedback loop, as shown by the substantial decrease on PAC1 staining observed in the presence of apyrase (**Figure 3H**). However, the thromboxane synthesis inhibitor indomethacin did not significantly affect platelet activation, whereas integrilin only inhibited PAC1 binding to integrin α_IIb_β_3_, but not α-granule secretion (**Figures 3G, H**). Finally, the combination of the three inhibitors (All3) did not promote additive effects on their effects (**Figures 3G, H**). These data demonstrate that feedback activation through ADP release mediates platelet function mediated by TLR2 agonists.

Next, we analyzed whether these inhibitors and blocking antibodies also affected TLT1 membrane expression. A similar pattern was observed as compared to P-selectin; namely, pharmacological inhibition of TLR2, NF-κB, and BTK caused a significant reduction of TLT1 exposure on the platelet membrane in response to Pam2CSK4 (**Supplementary Figure 1E**). However, the effect of secondary feedback inhibitors on the TLT1 exposure profile differed from that of P-selectin; in particular, indomethacin and integrilin significantly inhibited TLT1 exposure as measured by flow cytometry (**Supplementary Figure 1E**). In addition, using blocking antibodies to TLR2 and TLR6, we observed that Pam2CSK4-induced TLT1 exposure was also dependent on TLR2 and, to a lesser extent, on TLR6 signaling (**Supplementary Figure 1E**).

### TLR2 and ITAM Signaling Pathways Cooperate to Potentiate Platelet Activation

**Figure 4:**
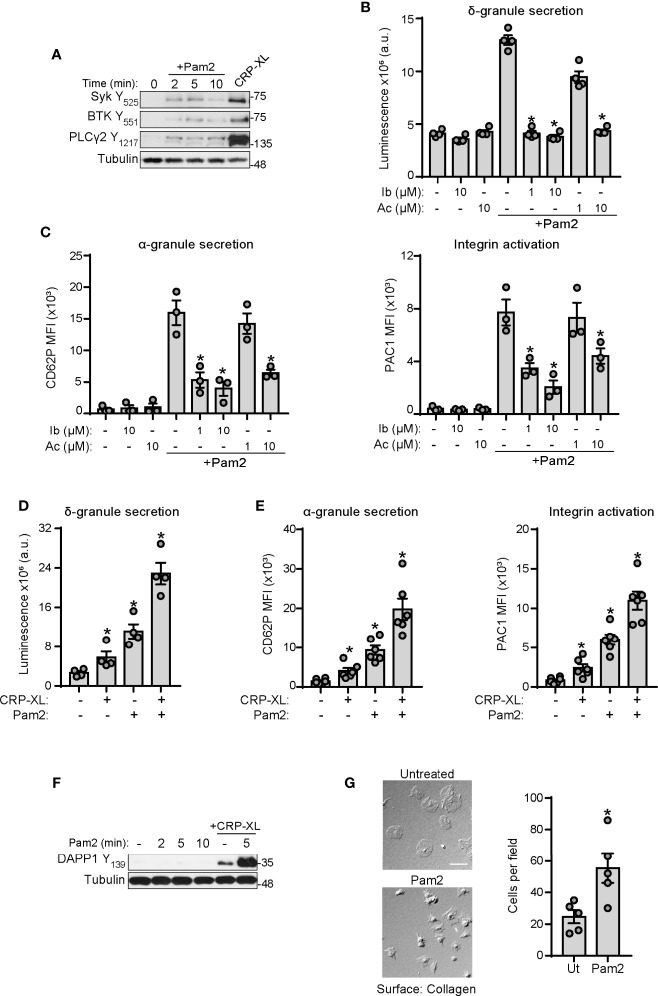
GPVI and TLR2 share common signaling nodes and cooperate to potentiate platelet activation. **(A)** Replicate preparations of washed human platelets (1x10^9^ ml) were activated with Pam2CSK4 (10 µg/ml) or CRP-XL (2 µg/ml; 5 min) and cell lysates analyzed by immunoblot for the indicated antibodies; n=3 independent experiments. **(B, D)** Platelets (2×10^8^/ml) were incubated with a luciferin luciferase reagent to measure ATP release and dense granule secretion. Data are expressed as raw mean luminescence (a.u.); n=4 experiments. **(C, E)** Platelets (5×10^7^/ml) were pre-treated and activated as above in the presence of fluorophore-conjugated antibodies against P-selectin (CD62P-APC) or the activated conformation of human integrin α_IIb_β_3_ (PAC1-FITC) and subsequent analysis by flow cytometry; n=3-6 experiments. Data are presented as total mean fluorescence intensity (MFI). **(F)** Platelet lysates were prepared as in A and analyzed by Western Blot for DAPP1 activation; representative of n=3 experiments. **(G)** Cover glass was coated with soluble collagen (50 μg/ml), blocked with BSA, and incubated with replicate samples of washed human platelets (2x10^7^/ml) untreated or stimulated with Pam2CSK4. After 45 min (37°C), cover glass was washed with PBS and adherent platelets were imaged with DIC microscopy; n=5 experiments. Pam2CSK4 was used at 10 µg/ml **(A–C, F, G)** or 5 µg/ml **(D, E)**. CRP-XL was used at 2 µg/ml. Scale bar indicates 10 µm. * indicates statistical significance (p<0.05) compared to Pam2CSK4 **(B, C)** or to untreated **(D–G)**. Error bars indicate standard error of the mean (SEM).

Our group has shown that BTK is a central player of platelet activation mediated by ITAM signaling (60, 61). Interestingly, BTK is also a well-known mediator of inflammation and immunity in many cells types, in part by mediating cellular responses to TLRs (62–64). Therefore, we asked whether BTK and other mediators of ITAM signaling also serve roles in platelet TLR2/TLR6 signaling. As shown in **Figure 4A**, stimulation of platelets with Pam2CSK4 increased phospholipase C (PLC)γ2 Y_1217_, BTK Y_551_ and Syk Y_535_ phosphorylation, although to a lesser extent than the positive control, CRP-XL. To further examine roles for BTK in platelet activation by Pam2CSK4, we preincubated platelets with BTK inhibitors ibrutinib and acalabrutinib, prior to Pam2CSK4 treatment. Preincubation of platelets with these inhibitors significantly reduced δ-granule secretion in response to Pam2CSK4 (**Figure 4B**), as well as α-granule secretion and integrin activation (**Figure 4C**). These results suggest that TLR2 and ITAM-mediated responses signal through overlapping nodes, where BTK regulates platelet responses to Pam2CSK4.

The activation of common signaling pathways downstream of the ITAM receptor GPVI and TLR2 suggested additive effects of these receptors on platelet function. Stimulation of platelets with combined suboptimal doses of CRP-XL and Pam2CSK4 enhanced platelet activation to a higher extent than both ligands alone, as shown by luminescence analysis of δ-granule secretion (**Figure 4D**) and flow cytometry analysis of P-selectin and PAC1 staining (**Figure 4E**). Signaling experiments failed to demonstrate a synergistic increase in the phosphorylation of main platelet signaling pathways such as Akt and PKC (data not shown). However, analysis of specific downstream mediators revealed that Pam2CSK4 is unable to activate dual adaptor of phosphotyrosine and 3-phosphoinositides 1 (DAPP1), but strongly potentiates DAPP1 activation mediated by CRP-XL (**Figure 4F**). To further confirm increased ITAM-mediated signaling by collagen in the presence of Pam2CSK4, we incubated Pam2CSK4-stimulated platelets onto collagen-coated surfaces. Activation of platelets with Pam2CSK4 significantly increased the number of platelets that adhere to collagen (**Figure 4G**). Altogether, these evidences suggest that the immune receptors GPVI and TLR2 are interconnected in platelets, and BTK is a common and targetable protein for platelet activation mediated by both receptors.

### Platelets Enhance Endothelial Cell Inflammation in Response to TLR2 Agonists

**Figure 5:**
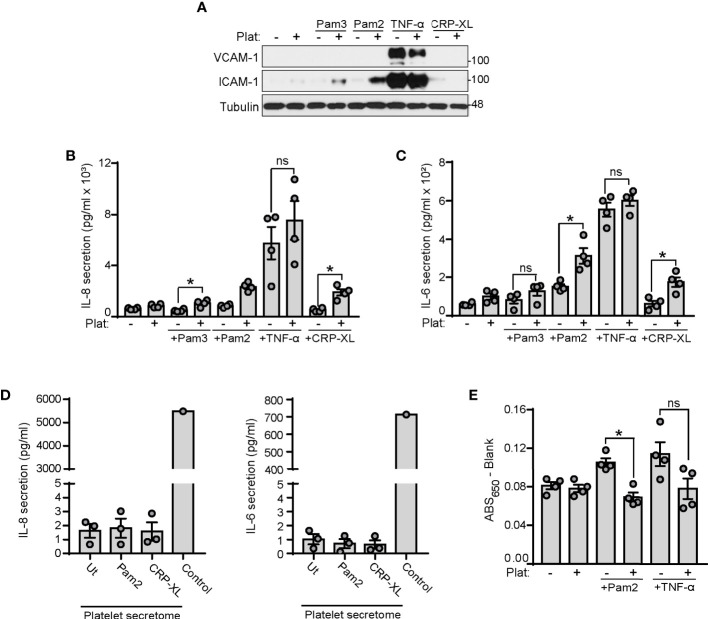
Platelets enhance endothelial cell inflammation and reduce endothelial permeability to BSA in response to TLR2 agonists. **(A–C)** HUVECs were cultured onto 12-well plates until reaching confluence and stimulated with Pam2CSK4 (10 µg/ml), Pam3CSK4 (10 µg/ml) or TNF-α (1 ng/ml). 250 µl of platelets (2x10^8^/ml) were mixed with 250 µl of SFM and co-cultured with HUVECs for 4 hours. **(A)** Adhered platelets were thoroughly removed with SFM and HUVECs lysates analyzed for ICAM-1 and VCAM-1 expression by immunoblot; n=4 experiments. **(B, C)** Supernatants were collected and assayed by ELISA for IL-6 and IL-8 secretion; n=4 experiments. **(D)** Washed platelets (1x10^9^/ml) were stimulated with Pam2CSK4 or CRP-XL at 10 µg/ml for 45 min at 37°C. Platelets were then pelleted and supernatants assayed for IL-6 and IL-8 secretion. A positive control from TNF-α stimulated HUVECs was used (Control); n=3. **(E)** HUVECs were seeded to confluence in transwell devices and stimulated for 4 h at 37°C with Pam2CSK4 (10 µg/ml) or TNF-α (1 ng/ml) in the presence or absence of platelets (1x10^8^/ml). Cells were washed 3 times and incubated with media containing Evans Blue dye (0.67 mg/ml) and 4% BSA for 30 min at 37°C. Samples from the lower chamber were collected and absorbance at 650 nm measured; n=4. * indicates statistical significance (p<0.05). ns indicates not significant. Plat indicates platelets. Error bars indicate standard error of the mean (SEM).

Platelets have roles in orchestrating leukocyte responses to TLR4 and TLR2 ligands (65–67). However, how platelets coordinate endothelial cell responses to TLR2 ligands remains unknown. Next, we stimulated HUVECs with the TLR2 ligands Pam2CSK4 and Pam3CSK4 in the presence or absence of platelets. TNF-α served as a positive control for inducing HUVECs inflammation and CRP-XL as positive control for platelet activation. Western blot analysis of adhesion molecules intercellular adhesion molecule-1 (ICAM-1) and vascular-cell adhesion molecule-1 (VCAM-1) showed that Pam2CSK4 and Pam3CSK4 did not induce ICAM-1 and VCAM-1 expression after 4 hours of stimulation in HUVECs (**Figure 5A**). However, when HUVECs were treated with Pam2CSK4 and Pam3CSK4 in the presence of platelets, the levels of ICAM-1 significantly increased, whereas VCAM-1 remained unaffected (**Figure 5A**). CRP-XL-treated platelets did not increase ICAM-1 expression in HUVECs. As interleukin (IL) secretion is another hallmark of endothelial cell inflammation, we analyzed IL-6 and IL-8 secretion in HUVECs supernatants. ELISA assays showed that Pam2CSK4 and Pam3CSK4 alone were too weak to induce IL secretion after 4 hours of treatment, but the presence of platelets significantly increased cytokine secretion when combined with Pam2CSK4 or Pam3CSK4 (**Figures 5B, C**). Of note, platelets did not increase HUVEC inflammation in response to 1 ng/ml TNF-α (**Figures 5A-C**). As a control, IL-6 and IL-8 were not increased in supernatants from Pam2CSK4-stimulated platelets, indicating that endothelial cell *de novo* synthesis causes the increased cytokine secretion (**Figure 5D**).

It has been demonstrated that stimulation with inflammatory agonists for 24 hours increases TLR2 expression on HUVECs membrane (68). Next, we asked whether platelets affect TLR2 expression on HUVECs in the presence of inflammatory agonists. As shown in **Supplementary Figure 2A**, Pam2CSK4 and TNF-α failed to significantly affect TLR2 expression on HUVECs after 4 hours of stimulation. Moreover, co-culture with platelets did not affect TLR2 expression in the presence of Pam2CSK4 or TNF-α.

In addition to regulating inflammation, platelets are also known to contribute to the regulation of vascular permeability (69, 70). To assess the role of platelet TLR2 activation in endothelial cell permeability, we performed a transwell permeability assay to quantify the rate of Evans Blue dye-stained BSA passage across a confluent layer of HUVECs. As shown in **Figure 5E**, treatment of HUVECs for 4 h with Pam2CSK4 increased their permeability to BSA; however, the presence of platelets reversed this effect, indicating that activation of platelets with Pam2CSK4 may help maintain ECs integrity in response to inflammatory insults. To note, platelets also reduced TNF-α-induced permeability in all experiments, although the data did not reach statistical significance due to experimental variability (**Figure 5E**). Altogether, these data suggest that activation of the platelet TLR2/TLR6 axis may trigger platelet-ECs interactions, which subsequently increases inflammation and reduces permeability in response to TLR2 agonists.

### Activation of Platelet TLR2/TLR6 Promotes Platelet Adhesion to HUVECs Under Static and Physiologically Relevant Flow Conditions

**Figure 6:**
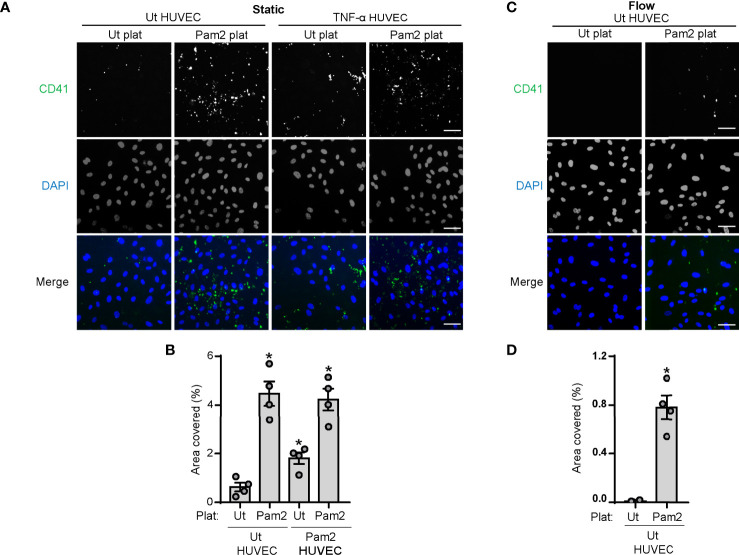
Stimulation with Pam2CSK4 increases platelet adhesion to HUVECs under static and flow conditions. HUVECs were seeded to confluence into Ibidi µ-Slide VI 0.1, serum starved for 2 h and stimulated with TNF-α at 1 ng/ml for 4 h when indicated. **(A–D)** Platelets (2x10^8^/ml) were stimulated with Pam2CSK4 (10µg/ml) and stained with a α-CD41 antibody (1:10) for 10 min at 37°C before co-culture with HUVECs. **(A, B)** For static assays, platelets (50 µl) where diluted 1:1 in SFM and incubated with HUVEC for 10 min at 37°C; n=4 experiments. **(C, D)** For flow adhesion experiments, platelets were flowed for 5 min at a flow rate of 18.69 µl/min; n=4 experiments. Cells were imaged using an inverted microscope with a 20X objective (Zeiss Axiovert 200M, Carl Zeiss). 5 random images were taken per field and data are presented as percentage of area covered by platelet CD41 staining. Scale bar indicates 50 µm. * indicates statistical significance (p<0.05) compared to untreated (Ut). Error bars indicate standard error of the mean (SEM).

Finally, we sought to determine whether activation of platelet TLR2/TLR6 by Pam2CSK4 modulates platelet adhesion to HUVECs monolayers under static or flow conditions. We stimulated platelets with Pam2CSK4 in the presence of a CD41-FITC antibody before incubation with HUVECs. Surface area analysis of CD41-stained platelets showed that Pam2CSK4 increases the amount of platelets adhered to both untreated and TNF-α-treated HUVECs monolayers under static conditions (**Figures 6A, B**). Moreover, pretreatment of HUVECs with Pam2CSK4 prior to performing the adhesion experiments did not affect the degree of platelet adhesion to HUVECs (**Supplementary Figures 2B, C**). We next performed adhesion assays under flow conditions flowing control and Pam2CSK4-stimulated platelets over unstimulated HUVECs in a microfluidic chamber. As shown in **Figures 6C, D**, the adhesion of unstimulated platelets to control HUVECs was almost inexistent under flow. However, preincubation of platelets with Pam2CSK4 significantly increased the number of platelets attached to HUVECs monolayers under flow (**Figures 6C, D**), which suggests that platelet TLR2/TLR6 activation may be sufficient to promote platelet-endothelial cell interactions.

## Discussion

**Figure 7:**
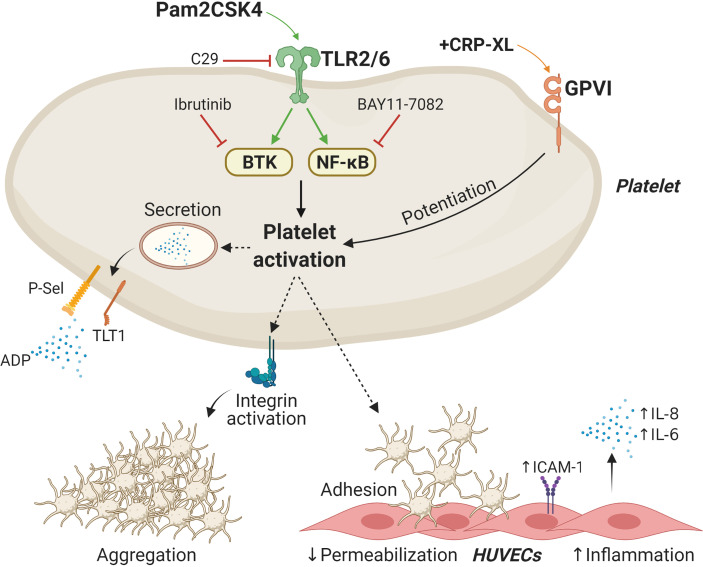
Schematic representation of Pam2CSK4 effects in platelets and platelet-HUVECs interactions. Activation of the TLR2/TLR6 complex by Pam2CSK4 initiates a series of signaling events involving BTK and NF-κB to promote platelet activation. Functional responses triggered by Pam2CSK4 include δ- and α-granule secretion as well as integrin activation and subsequent aggregation. Activation of platelet TLR2/TLR6 also promotes the adhesion of platelets to endothelial cells and increase endothelial cell inflammation. BTK, Bruton`s tyrosine kinase; HUVECs, Human umbilical vein endothelial cells; ICAM-1, intercellular adhesion molecule 1; TLT1, Trem-like transcript 1; NF-κB, nuclear factor-κB; GPVI, glycoprotein VI. Image created with BioRender.com.

Platelets express TLR2 signalosomes (31, 41), and TLR2/TLR1 ligands such as Pam3CSK4 have been established as potent platelet agonists (41). Here, we show that the synthetic diacylated lipoprotein Pam2CSK4, but not FSL-1, activates platelets and promotes platelet-HUVECs interactions (**Figure 7**). Mechanistic studies found that Pam2CSK4 induces granule secretion and integrin activation in a TLR2/NF-κB/BTK-dependent manner. Overall, our results suggest that platelets may become activated by PAMPs or DAMPs in inflammatory *milieus* and subsequently attach to vascular cells, therefore promoting increased inflammation. It is noteworthy that TLR2 may be activated not only by bacterial cell wall lipoproteins in the context of infection but also by endogenous molecules (DAMPs) in the context of sterile inflammation. Examples of DAMPs capable of activating TLR2 are heat-shock proteins, high mobility group box 1, biglycan, β-defensins and histones (71, 72). Our data suggest that dysregulated platelet TLR2 activation may promote increased platelet function and interactions with ECs in a manner supporting disease. The development of novel small-molecule inhibitors of TLRs would provide meaningful tools to further address the potential of these receptors as therapeutic targets in disease.

The immune and coagulation systems are tightly linked and regulate each other (73, 74). The terms “immunothrombosis” and “thromboinflammation” are used to define pro-thrombotic processes mediated by inflammatory mediators in conditions in which platelets and the coagulation system are pathologically activated (24, 25, 41). Examples of pathological platelet and coagulation activation in inflammation are sepsis and COVID-19 (24, 26). Interestingly, shedding of acylated lipopeptides from bacterial surfaces is an important mechanism for the initiation of the innate immune response and may contribute to thromboinflammation (75). Indeed, it has been demonstrated that lipoprotein detection by TLR2 contributes to the development of neonatal sepsis caused by Group B *Streptococcus* (76). Moreover, Group B *Streptococcus* strains found in septic patients can directly activate human platelets *via* TLR2 (77). In addition to infection, activation of platelet receptors has been suggested to play a role in promoting atherosclerosis and subsequent cardiovascular disease (78–80). Supporting a role for TLR2 in cardiovascular disease, TLR2 blockade promotes protective effects in endothelial cells *in vivo* and *in vitro* (81). Consequently, TLRs are gaining attention as potential therapeutic targets in both cardiovascular disease (5) and sepsis (82); however, the role of platelet TLRs in these conditions is poorly understood. Our study provides additional evidence that platelets may become activated in inflammatory contexts by specific lipoproteins targeting TLR2. Concretely, we demonstrate that a synthetic diacylated lipoprotein mimicking those found on certain bacterial walls, Pam2CSK4, triggers direct platelet activation *in vitro via* TLR2. In addition to purified systems, we also demonstrate that Pam2CSK4 induces platelet activation in whole blood, although we acknowledge that the underlying mechanisms in this experimental setup may be indirect as leukocytes also express TLR2/TLR6 (83). From a physiologic perspective, acylated lipoproteins are found in several pathogenic bacteria and may provide an additional link between the immune and coagulation systems and involve platelet activation. Consistent with our data, *in vivo* studies have demonstrated that diacylated lipoproteins are potent activators of TLR2 (84).

In this study we used three different TLR2 agonists. Consistent with previous reports (41), Pam2CSK4 induced a variable degree of aggregation, whereas Pam3CSK4 induced full aggregation for all the donors tested. Specific signal transduction events leading to differences in aggregation between Pam2CSK4 and Pam3CSK4 remain to be explored. Regarding differences between Pam2CSK4 and FSL-1, both are diacylated lipoproteins and recognized TLR2/TLR6 ligands in other cellular models (83). The structural differences between these ligands at their carboxyl terminal amino acids (CSK4 for Pam2CSK4 *vs* GDPKHPKSFK for FSL-1) may dictate their differential binding kinetics to the TLR2/TLR6 complex. Along this line, previous studies comparing TLR2/TLR6 ligands (including Pam2CSK4 and FSL-1) have demonstrated that these same agonists promote differential responses with varying kinetics in macrophages (83). These differences seem to be explained by the interaction of a particular peptide moiety within the ligands with the TLR2/TLR6 complex (85). Supporting this idea, a recent report demonstrated that the second and third amino acids of Pam2-like lipoproteins are key for promoting immune cell activation (86). The specific chemical groups mediating functional TLR2/TLR6 recognition in platelets remain to be identified, but the lack of activity of FSL-1 suggests that the CSK4 group is responsible of Pam2CSK4 activity. Another potential explanation for the lack of activity of FSL-1 is that it has been proposed as a potential TLR10 activator (87, 88). TLR10 is one of the less-studied TLRs, but recent reports suggest that it may be the only TLR promoting anti-inflammatory responses (87, 89). Interestingly, platelets express *TLR10* transcripts (18), although its expression at the protein level is yet to be demonstrated. Whether FSL-1 is able to target platelet TLR10 and counteract TLR2/TLR6 activation merits further investigation.

From an innate immunity perspective, platelets lack the expression of key receptor families such as interferon receptors, retinoid-inducible gene receptors or most nod-like receptors, yet they have conserved and express all TLRs (25, 45). This suggests that this group of ancient receptors may play a key role as platelet sensors of circulating inflammatory patterns present in infections (PAMPs) or in sterile inflammation (DAMPs). Elucidating the molecular mechanisms underlying TLR-mediated platelet activation may provide a means of identifying specific pathways regulating platelet activation in inflammation (90). Mechanistically, we demonstrate that TLR2 and TLR6 are involved in the binding and interaction of Pam2CSK4 with platelets. However, our data indicate that blockade of TLR2 with a C29 or TLR2 blocking antibody results in significant, but not complete, inhibition of platelet responses, which suggests that other receptor(s) may also be involved in the detection of Pam2CSK4 by platelets. A potential coreceptor for TLR2 is CD36. Supporting this hypothesis, Hoebe and colleagues initially demonstrated that signaling for select TLR2 ligands is dependent on CD36 (91). Subsequent studies demonstrated that the TLR2/TLR6 dimer is recruited to lipid rafts and forms a heterotypic association with CD36 to trigger downstream signaling events in response to diacylated lipopeptides, including Pam2CSK4 (92). More importantly, CD36 and the TLR2/TLR6 dimer have been shown to work in a synergistic manner to sense oxidized phospholipids by platelets (31). Based on this evidence, we hypothesize that CD36 may be a potential co-receptor for TLR2/TLR6 to sense Pam2CSK4 in platelets.

Additional pharmacologic experiments showed that NF-kB and MAPKs were involved in Pam2CSK4-induced responses, which is consistent with classical signaling downstream MyD88-dependent TLRs (2, 12, 54). Specific MAPKs activated included the family member p38, which is a well-known regulator of platelet functional responses in several contexts (93, 94). Akt was also activated in response to Pam2CSK4, which suggests that this pathway may be common for both TLR2/TLR1 and TLR2/TLR6 ligands (41). Another unexplored area on platelet activation by TLR2/TLR6 ligands was the potential involvement of classical platelet positive feedback loops (i.e. ADP and TxA2) (58). Experiments using the ADP scavenger apyrase demonstrated that the ADP positive feedback loop is necessary for both granule secretion and integrin activation mediated by Pam2CSK4. This evidence, together with the reported involvement of ADP in Pam3CSK4 responses (47), suggests that platelet activation by immune agonists is dependent on ADP loops in a similar manner as other classical platelet agonists. Interestingly, our data suggest that the mechanisms regulating P-selectin and TLT1 secretion seem to be differentially dependent on platelet secondary feedback loops, as indomethacin and integrilin significantly inhibited TLT1, but not P-selectin, exposure on the membrane following activation. This may explain the significant differences between TLT1 and P-selectin kinetics as potential markers of α-granule secretion suggested by previous studies (53). In addition to the mechanisms above, we also show that Pam2CSK4 signaling involves the activation of key nodes for the ITAM-receptor GPVI signaling pathway in platelets, namely PLCγ2, Syk and BTK (58). Our group has previously outlined the importance of BTK as a key node on regulating GPVI-driven platelet activation (60, 61). Some tyrosine kinase inhibitors targeting BTK, such as ibrutinib, have been shown to promote dysregulated platelet activation and bleeding in the clinical context (95). This work adds further evidence to the central role of BTK on platelet activation by demonstrating that the BTK inhibitors ibrutinib and acalabrutinib dampen platelet functional responses to Pam2CSK4. Consistent with our data, previous studies had shown activation of PLCγ2 and Syk in response to Pam3CSK4 in platelets, while ruled out that these effects were due to cross-activation of GPVI by Pam3CSK4 (42). These data suggest that TLR2/NF-κB may be targetable to reduce platelet activation in response to diacylated lipoproteins.

Platelets modulate leukocyte responses to TLR2 and TLR4 ligands (65, 66). In this line, studies have shown that platelet TLR2 mediates the formation of platelet-neutrophil aggregates (41) and platelet TLR4 supports neutrophil extracellular traps formation in inflammation (40). Moreover, platelet TLR2 and TLR4 are important for the formation of platelet-neutrophil aggregates in patients with essential thrombocythemia (96). In contrast, it remains unknown if platelet TLRs are capable of modulating endothelial cell responses to TLR2 agonists. HUVECs are known to express low amounts of TLR2 (97), but are still able to sense and respond to bacterial diacylated lipoproteins such as Pam3CSK4 (98). In our hands, Pam2CSK4 and Pam3CSK4 were not able to induce adhesion molecule expression and IL secretion after 4 h of treatment, but the presence of platelets significantly increased IL secretion and ICAM-1 expression in HUVECs in response to these agonists. As TLR2 expression on HUVECs remained unchanged in the presence of inflammatory agonists and platelets, our data suggest a role for platelet TLR2 on inducing increased endothelial cell inflammation in the presence of Pam2CSK4. The mechanisms underlying increased HUVECs inflammation in the presence of platelets remained to be defined, but may be due to the release of inflammatory mediators by platelets or by direct attachment of platelet membrane receptors to specific receptors on HUVECs. The classical platelet agonist CRP-XL also induced a significant increase on IL secretion in the presence of platelets, but it did not affect ICAM-1 expression. This suggests that there may be subtle differences on how platelets regulate endothelial inflammation upon stimulation with classical agonists such as CRP-XL and inflammatory ligands such as Pam2CSK4. In addition to inflammation, platelets are also known to regulate vascular permeability (69, 70, 99). Our transwell experiments show that activation of platelet TLR2 reduces endothelial permeability to BSA upon stimulation with Pam2CSK4. This is consistent with a previous study that reported decreased permeability of pulmonary endothelial cells to albumin in the presence of platelets (99). Overall, our data suggest that activation of platelet TLR2 may modulate vascular function not only by enhancing inflammation and immune cell recruitment, but also by reducing vascular permeability in response to inflammatory insults.

An important field of study in cardiovascular disease is the adhesion of circulating cells, including platelets, to the vessel walls, which may promote disease progression in certain contexts (78). It is widely accepted that inflamed endothelium supports the adhesion of circulating platelets, mainly *via* expression of Von Willebrand Factor under flow conditions (100). Additional *in vivo* studies have shown that platelets roll over stimulated endothelium in a manner regulated by P-selectin (101). The adhesion of platelets to stimulated endothelium is also supported by *in vitro* studies demonstrating that platelets adhere to thrombin-treated endothelial cells (102). Interestingly, platelets are able to adhere not only to vascular endothelial cells but also to their valvular counterparts *in vitro* (103). On the other hand, whether activated platelets are capable of adhering to unstimulated endothelium remains controversial. In this study we provide additional evidence to the interactions between platelets and venous ECs, showing that activation of platelet TLR2/TLR6 with Pam2CSK4 is sufficient to promote platelet adhesion to untreated HUVECs monolayers under both static and physiologically relevant flow conditions. It is noteworthy that Pam2CSK4-activated platelets adhered to a similar extent to untreated or TNF-α-treated HUVECs under static conditions. This suggests that activation of platelets plays a more prominent role than activation of ECs in mediating adhesion. Consistent with our results, it has been reported that activated platelets can bind to intact endothelium (104), and other study showed that thrombin-activated platelets adhere to untreated endothelium (105). However, it remains unclear whether these interactions are transient or able to modify endothelial cell phenotype in *in vivo* conditions.

It should be noted that this study presents a series of limitations. Whereas our data show robust activation of platelets *in vitro* and *ex vivo* by Pam2CSK4, whether these mechanisms occur *in vivo* in the setting of bacterial infections or other disease contexts remains to be explored. Moreover, future studies are needed to elucidate whether platelets modulate TLR and inflammatory molecule expression in HUVECs at time points greater than 4 hours. Finally, our ECs experiments are restricted to ECs from venous origin (HUVECs), which are knows to express low TLR2 and TLR6 levels. Thus, it remains to be seen how our findings will translate to endothelial cell types which express higher levels of TLR2 and TLR6, such as arterial ECs or lung microvascular ECs.

## Conclusions

Platelet TLR2 can be activated by specific diacylated lipopeptides to promote robust platelet activation and platelet-endothelial cell interactions. This work set the basis to explore the potential of the TLR2/TLR6 axis as targets to dampen excessive platelet activation and inflammation in inflammatory conditions.

## Data Availability Statement

The raw data supporting the conclusions of this article will be made available by the authors, without undue reservation.

## Author Contributions

Concept and design: IP-I and JA. Data interpretation: all authors. Manuscript drafting: IP-I, JP, OM, and JA. Obtained funding: OM and JA. Study supervision: OM and JA. All authors contributed to the article and approved the submitted version.

## Funding

This work was supported by the American Society of Hematology (ASH Scholar Award to JA), the National Institutes of Health (R01HL146549 to JA and R01HL101972 and R01AI57037 to OM), and the Medical Research Foundation of Oregon.

## Conflict of Interest

The authors declare that the research was conducted in the absence of any commercial or financial relationships that could be construed as a potential conflict of interest.

## Publisher’s Note

All claims expressed in this article are solely those of the authors and do not necessarily represent those of their affiliated organizations, or those of the publisher, the editors and the reviewers. Any product that may be evaluated in this article, or claim that may be made by its manufacturer, is not guaranteed or endorsed by the publisher.

## Supplementary Material

Supplementary Figure 1 Dose-response for FSL-1 and mechanisms regulating TLT1 exposure on the membrane. (A, B, E) Washed platelets (5´107/ml) were activated with the indicated ligands in the presence of fluorophore-conjugated antibodies against P-selectin (CD62P-APC), TLT1 (TLT1-FITC) or the activated conformation of human integrin aIIbb3 (PAC1-FITC) at 1:25 and subsequently analyzed by flow cytometry. CRP-XL was used as positive control (10 μg/ml). **(A, B)** Dose response for FSL-1; n=3 independent experiments. **(C, D)** Citrated whole blood was diluted 1:5 in HT and activated with FSL-1 at the indicated doses or CRP-XL at 2 µg/ml. CD62P-APC and PAC1-FITC staining was measured in the platelet gate; n=3 independent experiments. **(E)** Platelets were preincubated with the indicated inhibitors for 10 min or blocking antibodies for 30 min at 37°C prior to activation with Pam2CSK4 at 10 μg/ml and measurement of TLT1 exposure on the membrane; n=3 independent experiments. C29 was used at 40 µM. BAY indicates BAY11-7082 10 μM; Ib, 10 μM Ibrutinib; Ac, 10 µM Acalabrutinib; Ap, 2 U/ml apyrase; In, 10 mM indomethacin; Int, 20 mg/ml integrilin. Data are presented as total mean fluorescence intensity (MFI). * indicates statistical significance (p < 0.05) compared to untreated (Ut) **(A–D)** or to Pam2 (-) **(E)**. Error bars indicate standard error of the mean (SEM).

Supplementary Figure 2 Platelets do not affect TLR2 expression on HUVECs and stimulation of HUVECs with Pam2CSK4 does not affect platelet adhesion. (A) HUVECs were cultured onto 6-well plates until reaching confluence and stimulated with Pam2CSK4 (10 μg/ml) or TNF-α (1 ng/ml) in the presence or absence of platelets (2x108/ml) for 4 h at 37°C. Then, cells were resuspended and stained with a TLR2-FITC antibody. Samples analyzed with a BD FACSCanto II flow cytometer. Data are presented as mean fluorescence intensity (MFI). **(B, C)** HUVECs were seeded to confluence into Ibidi μ-Slide VI 0.1, serum starved for 2 h and stimulated with Pam2CSK4 at 10 µg/ml for 4 h when indicated. Platelets (2x108/ml) were unstimulated or stimulated with Pam2CSK4 (10µg/ml) and stained with a α-CD41 antibody (1:10) for 10 min at 37°C before co-culture with HUVECs. Cells were imaged using an inverted microscope with a 20X objective (Zeiss Axiovert 200M, Carl Zeiss). 5 random images were taken per field (representative images shown in A) and data are presented as percentage of area covered by platelet CD41 staining **(B)**. Scale bar indicates 50 μm. * indicates statistical significance (p < 0.05) compared to untreated (Ut, Ut HUVEC). Error bars indicate standard error of the mean (SEM).

## Abbreviations

a.u., arbitrary units; BSA, bovine serum albumin; BTK, Bruton’s-tyrosine kinase; CRP-XL, (cross-linked) collagen-related peptide; DAMPs, damage-associated molecular patterns; DIC, Kohler-illuminated Nomarski differential interference contrast microscopy; ECs, Endothelial cells; GPVI, glycoprotein VI; HT, HEPES-Tyrode buffer; HUVECs, Human umbilical vein endothelial cells; IL, interleukin; ITAM, immune receptor tyrosine-based activation motif; ICAM-1, Intercellular adhesion molecule-1; LPS, Lipopolysaccharide; MAPK, Mitogen-activated protein kinases; MFI, mean fluorescence intensity; MyD88, myeloid Differentiation primary response 88; PAMPs, pathogen-associated molecular patterns; PRR, pattern-recognition receptor; PBS, phosphate buffered saline; PFA, paraformaldehyde; PGI_2_, prostaglandin I2; PRP, platelet rich plasma; rt, room temperature; TLRs, Toll-like-receptors; VCAM-1, Vascular-cell adhesion molecule-1.
