# Supplementary figures and images for: The Toll-Like Receptor 2 Ligand Pam2CSK4 Activates Platelet Nuclear Factor-κB and Bruton’s Tyrosine Kinase Signaling to Promote Platelet-Endothelial Cell Interactions
Source: Front Immunol. 2021 Aug 30;12:729951. doi: 10.3389/fimmu.2021.729951 (PMC8435771; doi:10.3389/fimmu.2021.729951)

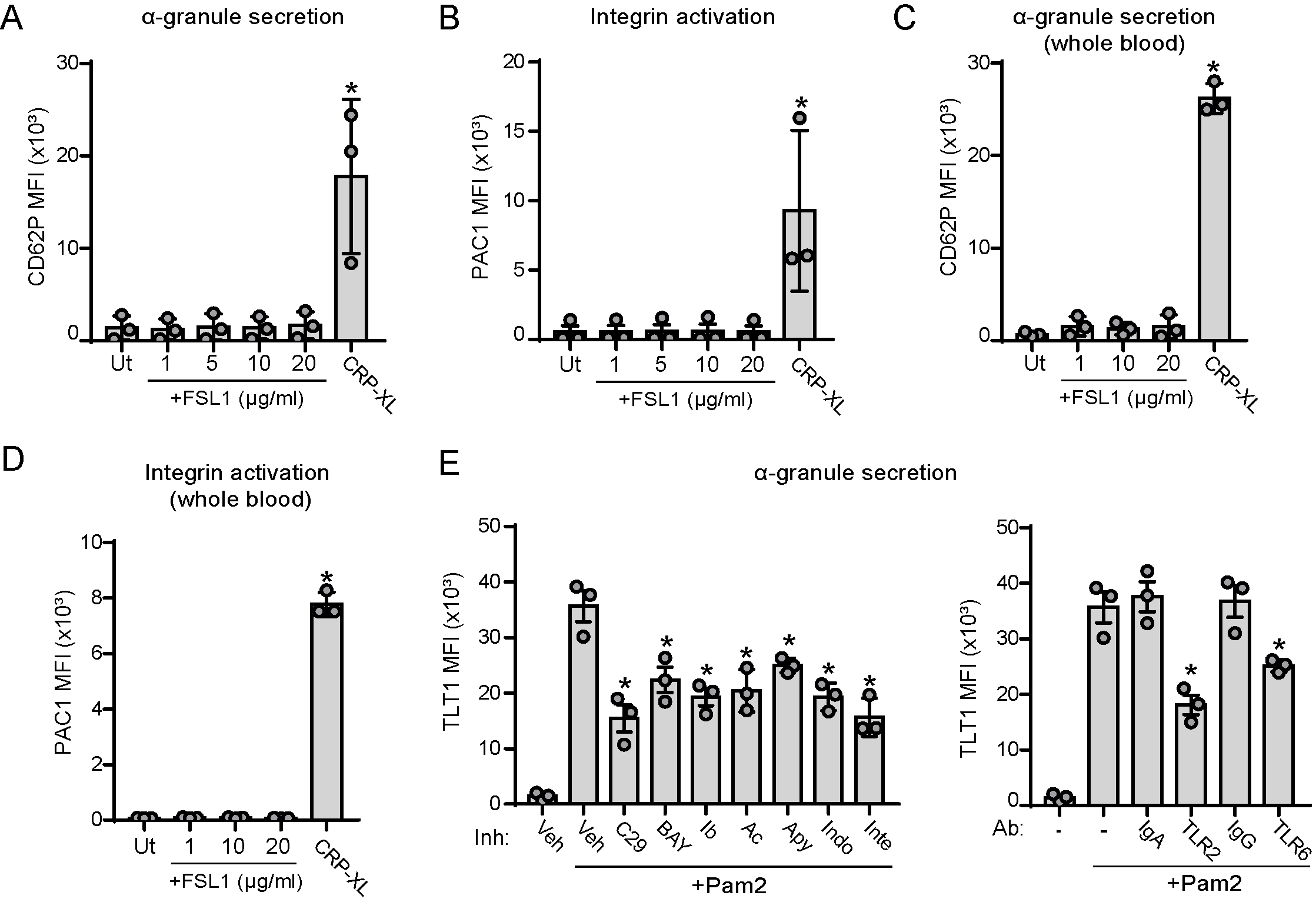

Supplement: Supplementary Figure 1 — Dose-response for FSL-1 and mechanisms regulating TLT1 exposure on the membrane. (A, B, E) Washed platelets (5´107/ml) were activated with the indicated ligands in the presence of fluorophore-conjugated antibodies against P-selectin (CD62P-APC), TLT1 (TLT1-FITC) or the activated conformation of human integrin aIIbb3 (PAC1-FITC) at 1:25 and subsequently analyzed by flow cytometry. CRP-XL was used as positive control (10 μg/ml). (A, B) Dose response for FSL-1; n=3 independent experiments. (C, D) Citrated whole blood was diluted 1:5 in HT and activated with FSL-1 at the indicated doses or CRP-XL at 2 µg/ml. CD62P-APC and PAC1-FITC staining was measured in the platelet gate; n=3 independent experiments. (E) Platelets were preincubated with the indicated inhibitors for 10 min or blocking antibodies for 30 min at 37°C prior to activation with Pam2CSK4 at 10 μg/ml and measurement of TLT1 exposure on the membrane; n=3 independent experiments. C29 was used at 40 µM. BAY indicates BAY11-7082 10 μM; Ib, 10 μM Ibrutinib; Ac, 10 µM Acalabrutinib; Ap, 2 U/ml apyrase; In, 10 mM indomethacin; Int, 20 mg/ml integrilin. Data are presented as total mean fluorescence intensity (MFI). * indicates statistical significance (p < 0.05) compared to untreated (Ut) (A–D) or to Pam2 (-) (E). Error bars indicate standard error of the mean (SEM). [file DataSheet_1.zip › Supplementary Figure 1.tif]

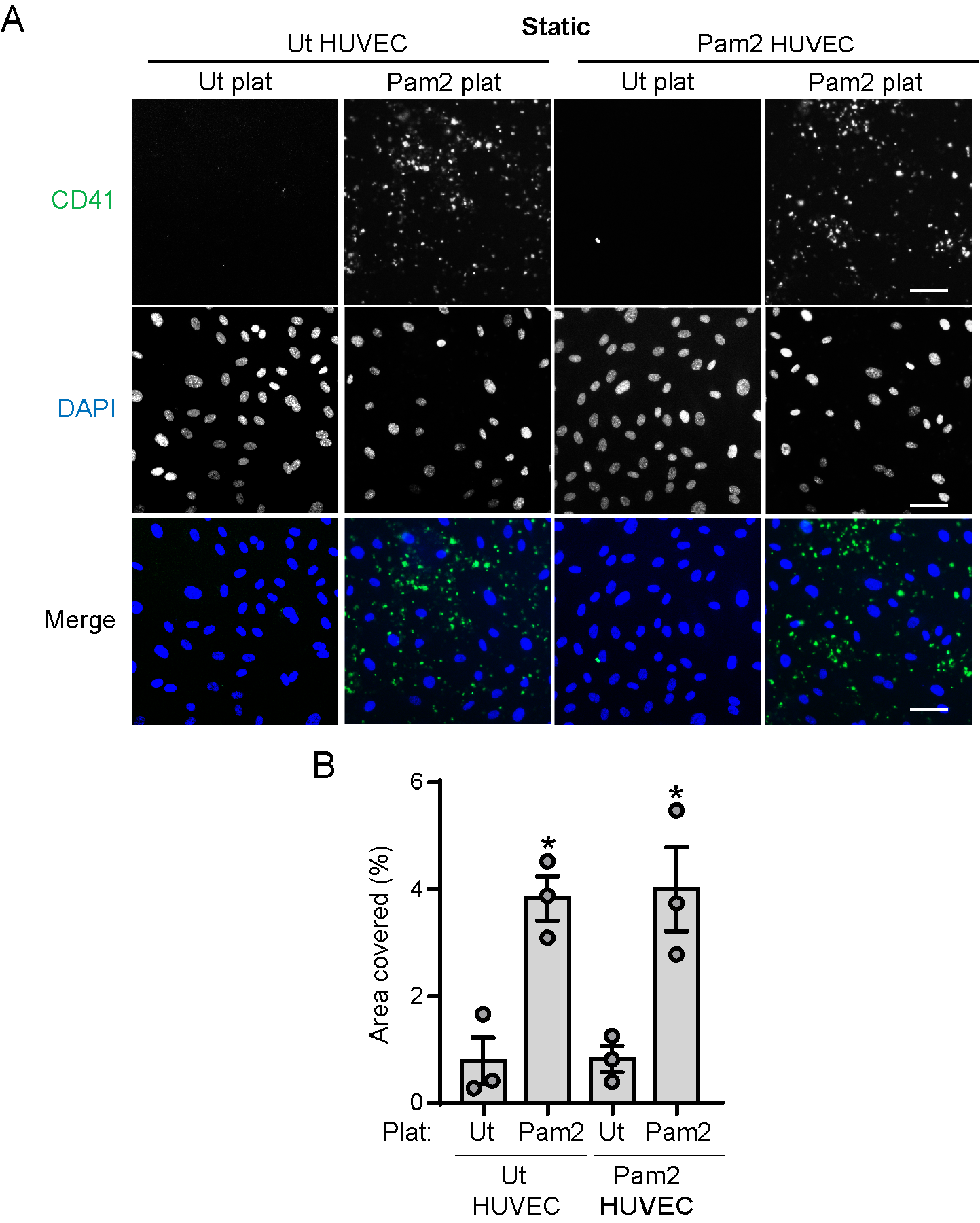

Supplement: Supplementary Figure 1 — Dose-response for FSL-1 and mechanisms regulating TLT1 exposure on the membrane. (A, B, E) Washed platelets (5´107/ml) were activated with the indicated ligands in the presence of fluorophore-conjugated antibodies against P-selectin (CD62P-APC), TLT1 (TLT1-FITC) or the activated conformation of human integrin aIIbb3 (PAC1-FITC) at 1:25 and subsequently analyzed by flow cytometry. CRP-XL was used as positive control (10 μg/ml). (A, B) Dose response for FSL-1; n=3 independent experiments. (C, D) Citrated whole blood was diluted 1:5 in HT and activated with FSL-1 at the indicated doses or CRP-XL at 2 µg/ml. CD62P-APC and PAC1-FITC staining was measured in the platelet gate; n=3 independent experiments. (E) Platelets were preincubated with the indicated inhibitors for 10 min or blocking antibodies for 30 min at 37°C prior to activation with Pam2CSK4 at 10 μg/ml and measurement of TLT1 exposure on the membrane; n=3 independent experiments. C29 was used at 40 µM. BAY indicates BAY11-7082 10 μM; Ib, 10 μM Ibrutinib; Ac, 10 µM Acalabrutinib; Ap, 2 U/ml apyrase; In, 10 mM indomethacin; Int, 20 mg/ml integrilin. Data are presented as total mean fluorescence intensity (MFI). * indicates statistical significance (p < 0.05) compared to untreated (Ut) (A–D) or to Pam2 (-) (E). Error bars indicate standard error of the mean (SEM). [file DataSheet_1.zip › Supplementary Figure 2.tif]
